# Comparison between OLIF and MISTLIF in degenerative lumbar stenosis: an age-, sex-, and segment-matched cohort study

**DOI:** 10.1038/s41598-023-40533-7

**Published:** 2023-08-14

**Authors:** Lantao Liu, Hui Xue, Zhiyuan Han, Lianghai Jiang, Longwei Chen, Dechun Wang

**Affiliations:** 1 Department of Spinal Surgery, Qingdao Municipal Hospital, Donghai Zhong Road No. 5, Qingdao, 266000 Shandong People’s Republic of China; 2 Graduate School of Dalian Medical University, No. 9 West Section of Lushun South Road, Dalian, 116044 Liaoning People’s Republic of China

**Keywords:** Bone, Spine regulation and structure

## Abstract

To compare outcomes after oblique lateral interbody fusion (OLIF) versus minimally invasive transforaminal lumbar interbody fusion (MISTLIF) with bilateral decompression via unilateral approach for treating mild to moderate symptomatic degenerative lumbar spinal stenosis (DLSS). We retrospectively compared patients who underwent single-level (L4/5) OLIF with an age-, sex-, and segment-matched MISTLIF with bilateral decompression via unilateral approach cohort. Perioperative data were collected for the operative time, intraoperative blood loss, drainage in the first postoperative day, postoperative hospital stay, cost, intraoperative fluoroscopy, and complications. Lumbar radiographs were measured for changes in posterior intervertebral space height (PISH), intervertebral space foramen height (IFH), intervertebral foramen area (IFA), and area of the spinal canal (ASC). Clinical and psychological outcomes included the visual analog scale (VAS), Oswestry Disability Index (ODI), and hospital anxiety and depression scale (HADS). 35 OLIF patients were compared with 35 MISTLIF patients in L4/5 DLSS. The OLIF group had shorter bedtime, postoperative hospital stays, less intraoperative and postoperative blood loss (all *P* < 0.05), but had more times of intraoperative fluoroscopy, longer operative time, and higher cost (all *P* < 0.05). The complication rates were equivalent (OLIF vs MISTLIF: 22.86% vs 17.14%). PISH (11.94 ± 1.78 mm vs 9.42 ± 1.94 mm, *P* < 0.05), IFH (23.87 ± 3.05 mm vs 21.41 ± 2.95 mm, *P* < 0.05), and IFA (212.14 ± 51.82 mm^2^ vs 177.07 ± 51.73 mm^2^, *P* < 0.05) after surgery were significantly increased in the OLIF group. The ASC was increased significantly after the operation in both groups, but the ASC in the MISTLIF group was increased significantly more than that in the OLIF group (450.04 ± 66.66 mm^2^ vs 171.41 ± 58.55 mm^2^, *P* < 0.05). The lumbar VAS scores at 1 month (1.89 ± 0.87 vs 2.34 ± 0.84, *P* = 0.028) and 6 months (1.23 ± 0.97 vs 1.80 ± 0.99, *P* = 0.018) after operation in the OLIF group were significantly lower. There were no significant differences in lower extremity VAS and ODI scores between the two groups. Compared with MISTLIF group, HADS scores on postoperative day 3 (2.91 ± 1.46 vs 4.89 ± 1.78, *P* < 0.05) and prior to hospital discharge (PTD) (2.54 ± 1.38 vs 3.80 ± 1.78, *P* = 0.002) in the OLIF group were decreased significantly. OLIF showed more advantages of less surgical invasion, lower incidence of postoperative low back pain, faster postoperative recovery, and less anxiety compared with MISTLIF. Regardless of cost, OLIF seems to be a better option to treat mild to moderate symptomatic DLSS.

## Introduction

Degenerative lumbar spinal stenosis (DLSS), which mostly occurred in elderly patients over the age of 65 years, is one of the main causes of low back pain and lower extremity dysfunction^1^. When the conservative treatment is ineffective or the symptoms are further aggravated, decompression and fusion become necessary treatment. The first-hand systematic evidence from the Swedish Lumbar Spine Study Group (SLSSG) suggests that lumbar fusion surgery has better clinical outcomes concerning reducing symptoms and improving limb function than nonsurgical treatment, and the cost is less expensive than conservative treatment in long-term follow-up^2,3^. Therefore, lumbar interbody fusion is a determined treatment for lumbar degenerative disease^4^.

Posterior lumbar interbody fusion (PLIF) and transforaminal lumbar interbody fusion (TLIF) are common surgical methods for the treatment of lumbar spinal stenosis. Traditional PLIF is limited by causing iatrogenic injury to the paraspinal musculature, over dural extraction, and disruption of the posterior tension band. In recent years, TLIF has been the most commonly used to treat DLSS. TLIF can obtain a good decompression effect and maintain satisfactory spinal stability by resecting the bilateral facet joints and part of the lamina, loosening surrounding fibrous tissue, and implanting pedicle screws and intervertebral cage. TLIF also can be performed via a minimally invasive surgical approach. Although minimally invasive transforaminal lumbar interbody fusion (MISTLIF) can reduce the injury to the posterior branch of the spinal nerve, reduce the denervation of multifidus muscle and reduce the incidence of intramuscular degeneration and necrosis^5^, patients undergoing MISTLIF often suffer from chronic back pain after surgery, which may be due to the resection of paravertebral muscle and facet joints^6^. The surgical method that can maximize the preservation of facet joints and reduce paravertebral muscle injury is more helpful to reduce complications. Thus, more and more surgeons have been seeking minimally invasive surgical methods for DLSS. MISTLIF with bilateral decompression via unilateral approach can not only obtain good clinical effect but also preserve the contralateral articular process and reduce the incidence of complications^7^.

Oblique lateral interbody fusion (OLIF), another minimally invasive surgery, has recently been adopted by spine surgeons as a minimally invasive and rigid fixation procedure for degenerative disc diseases (DDD). In OLIF surgery, spine surgeons insert a large cage between the vertebrae to achieve indirect decompression through the retroperitoneal space via the interval between the psoas muscle and major vessel assisted by the retractor^8^. OLIF was reported with satisfactory efficacy in lumbar degenerative diseases in cohort studies^9^; however, rare studies compared its efficacy with MISTLIF surgery with bilateral decompression via a unilateral approach in DLSS^10^. The present study evaluates the radiographic and clinical outcomes after OLIF in comparison to an age-, sex-, and segment-matched cohort of patients undergoing bilateral decompression via a unilateral approach MISTLIF for the mild to moderate symptomatic DLSS.

## Materials and methods

### Ethics statement

This study was approved by the institutional review board of the ethics committee of Qingdao Municipal Hospital and was conducted in accordance with the Declaration of Helsinki. It was a retrospective cohort study comparing patients under-going single-level OLIF versus MISTLIF with bilateral decompression via a unilateral approach at a single center. The 2 cohorts were matched by sex, age, and segment. All the participants gave informed consent before taking part. The datasets analysed during the current study are not publicly available due privacy of the patients but are available from the corresponding author on reasonable request.

### Patients

**Figure 1:**
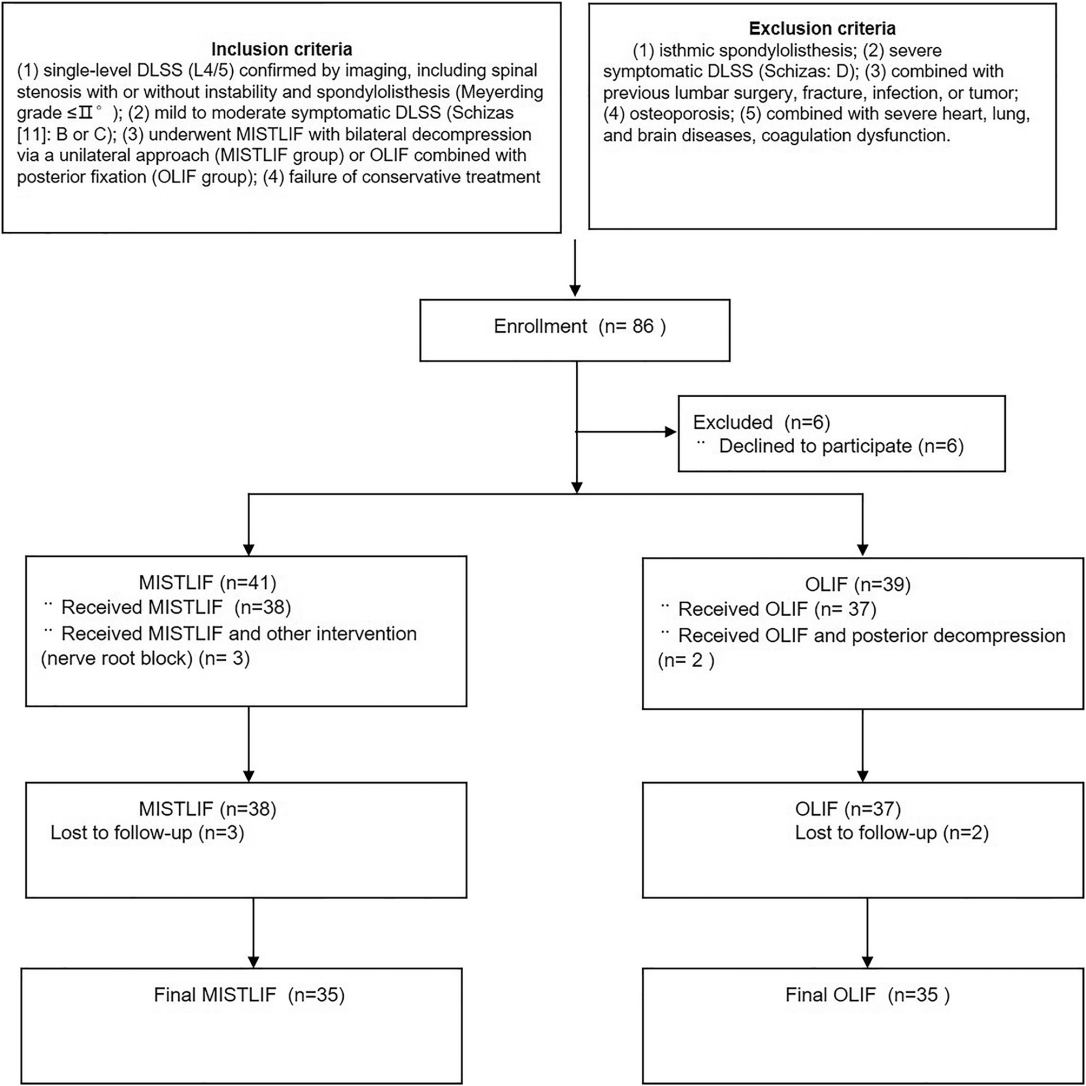
Flow chart for patient population, grouping, intervention and follow-up method.

We finally retrospectively compared 35 patients who underwent single-level (L4/5) OLIF with an age-, sex-, and segment-matched MISTLIF with bilateral decompression via unilateral approach cohort from November 2018 to January 2021 (Fig. 1). The main basis for choosing the surgical method is as follows: (1) If the working corridor (distance between aorta and psoas major) allows, we prefer OLIF surgery, and if the working corridor does not allow, we choose MISTLIF. (2) MISTLIF and OLIF were utilized at different times. Early on, MISTLIF was mostly used to treat lumbar degenerative diseases, but OLIF was primarily used to treat them later on. Inclusion criteria: (1) single-level DLSS (L4/5) confirmed by imaging, including spinal stenosis with or without instability and spondylolisthesis (Meyerding grade ≤ II°); (2) mild to moderate symptomatic DLSS (Schizas^11^: B or C); (3) underwent MISTLIF with bilateral decompression via a unilateral approach (MISTLIF group) or OLIF combined with posterior fixation (OLIF group); (4) failure of conservative treatment. Exclusion criteria: (1) isthmic spondylolisthesis (2) severe symptomatic DLSS (Schizas: D); (3) combined with previous lumbar surgery, fracture, infection, or tumor; (4) osteoporosis; (5) combined with severe heart, lung, and brain diseases, coagulation dysfunction. All the patients were followed up at least for 12 months (ranges: 12 months to 24 months).

### Surgical procedure

#### MISTLIF group

Each patient underwent tracheal intubation under general anesthesia. After the appropriate quadrant expandable channel was installed and the segment was correct, the soft tissue of the lamina and the facet surface were removed. Under the microscope, the unilateral lamina, superior and inferior articular processes, and intervertebral disc were removed, then the unilateral spinal canal was decompressed. After that, an appropriately sized interbody fusion cage was placed. The pedicle screw and the connecting rod were placed on the decompression side. The operating bed was tilted to the opposite side, and the magnification and field of view of the microscope were adjusted. The base of the spinous process and the contralateral part of the lamina was blurred away under the microscope. A laminator was used to dissect the ligament until the contralateral never root was revealed. The pedicle screw was fixed under the channel on the opposite side. Wound drainage was applied in all cases.

#### OLIF group

The procedure of OLIF was performed as reported by Fan^12^. In brief, the patient took the right lying position after general anesthesia. A 4-cm long incision was made 3.0–5.0 cm forward of the midpoint of the target intervertebral disc. Then separate the layers of abdominal muscles along the direction of the external oblique, the internal oblique, and the transverse abdominal muscle, respectively, and expose the retroperitoneal space. A working channel was placed and fixed after the correct surgical segment was determined with the guide needle by the C-arm X-ray. The target segment intervertebral disc was removed, then the cartilage endplate was scraped. A cage of appropriate height and length, which was filled with artificial bone, was knocked into the intervertebral space. After the position of the cage was confirmed well by the C-arm, close the incision layer by layer. Adjust the patient to a prone position and perform posterior internal fixation with pedicle screw instrumentation. Whether to place the drainage tube in the surgical area according to the bleeding condition in the operation.

### Perioperative parameters

The perioperative clinical data of the patients were recorded, including the operative time, intraoperative blood loss, drainage in the first postoperative day, postoperative bedtime, hospital stay, cost (total cost and high-value medical consumables cost), intraoperative fluoroscopy, and complications. Transient thigh flexion weakness is usually occurred in OLIF surgery and refers to leg weakness or numbness that was only temporary in nature and went away during the first three months, even if surgery was not necessary.

### Radiographic parameters

**Figure 2:**
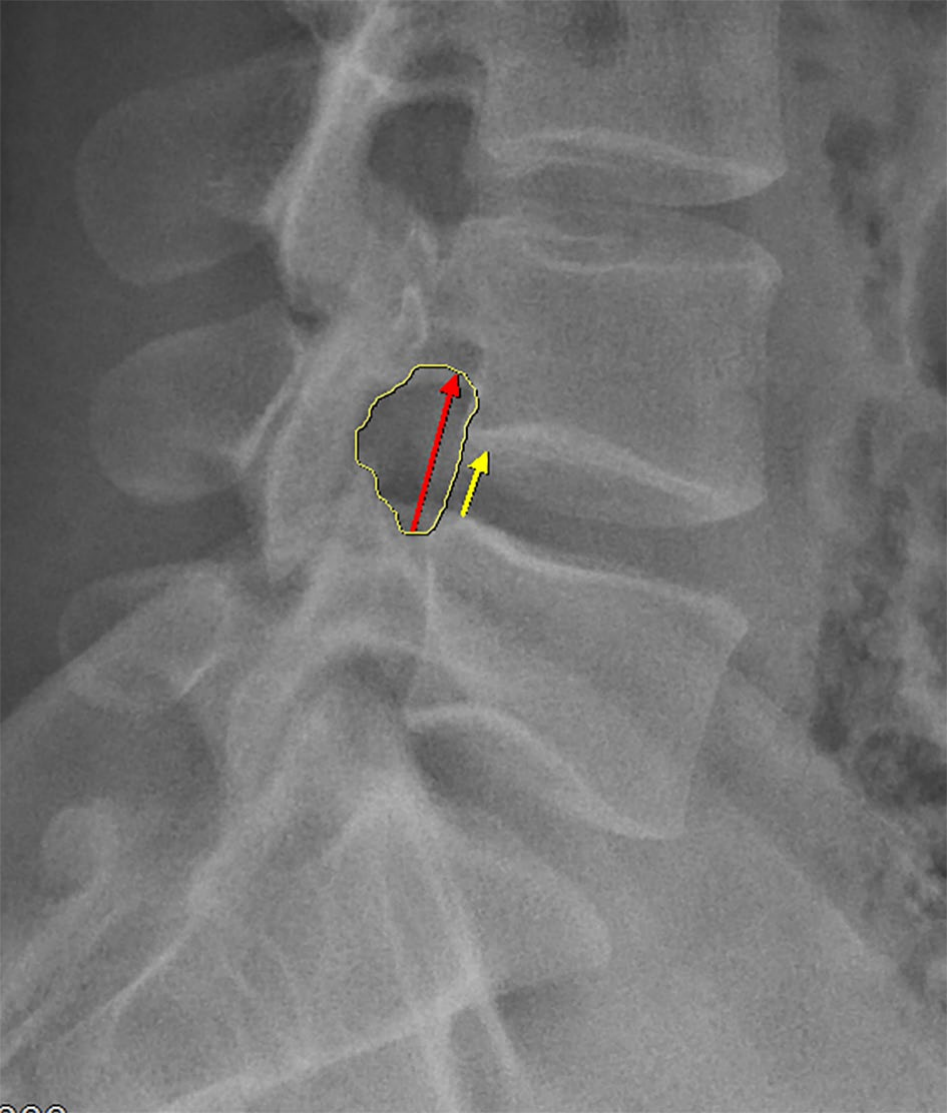
Overview of the radiographic parameters of interest. Yellow arrow indicated PISH; red arrow indicated IFH; yellow area indicated IFA.

**Figure 3:**
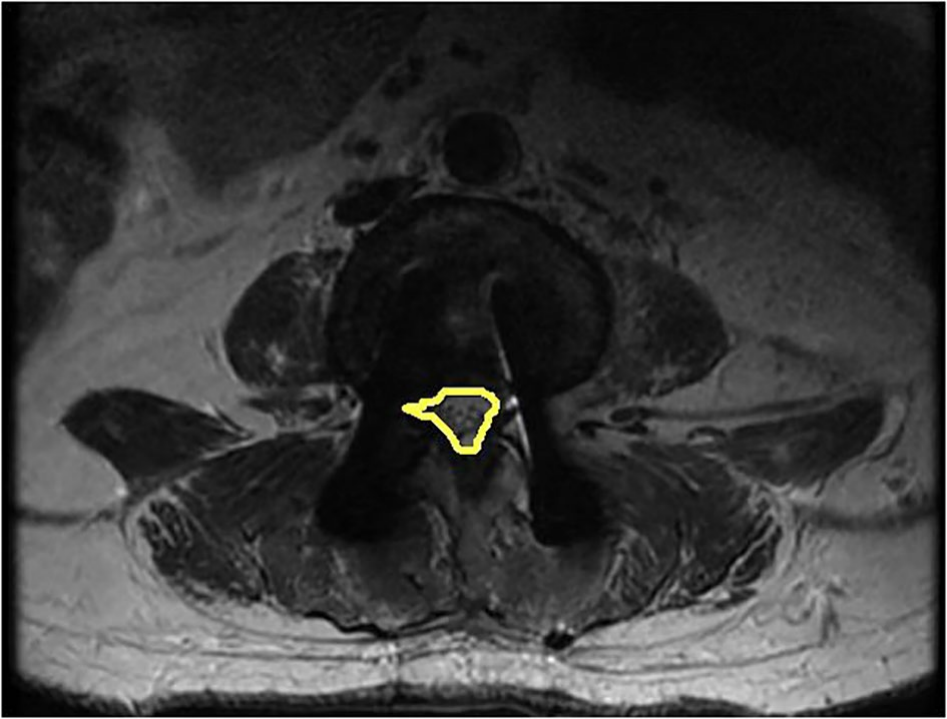
Measurement of the radiographic parameters of ASC.

The imaging evaluation was performed using a PAC system. Posterior intervertebral space height (PISH), intervertebral space foramen height (IFH), and intervertebral foramen area (IFA) were collected from X-ray film. The PISH was measured as distance between posterior margin of inferior endplate of the L4 vertebra and posterior margin of superior endplate of the L5 vertebra on lateral radiograph. The IFH was measured as the longest distance between lower margin of the L4 pedicle and the upper margin of L5 pedicle on lateral radiograph. IFA was defined as the region enclosed by the postero-inferior portion of the superior vertebral body, the superior and inferior adjacent vertebral pedicles, the ligamentum flavum surface anteriorly, the postero-superior portion of the inferior vertebral body, the surface of the intervertebral disk posteriorly, and the superior and inferior adjacent vertebral pedicles. The measurement methods of PISH, IFH, and IFA were shown in Fig. 2. The area of the spinal canal (ASC) was calculated from the mean value of the three different slice areas from T2 magnetic resonance imaging (MRI) in the axial view (Fig. 3).

### Clinical and psychological outcomes

Clinical outcomes were assessed using the visual analog scale (VAS), and Oswestry Disability Index (ODI) preoperatively, at 1, 6, 12 months, and final follow-up. The hospital anxiety and depression scale (HADS)^13^ was used to evaluate the psychological outcomes of patients preoperatively, at 3 days after the operation, and prior to hospital discharge (PTD). The final follow-up was defined as the periods between more than 12–24 months.

### Statistical methods

Statistical analysis of the data was performed using IBM SPSS 20.0 statistical software (International Business Machines Corporation, Armonk, New York, USA). Continuous or discrete variables are presented as mean ± standard deviation (SD), and categorical variables are expressed as frequency or percentage. Data were screened for abnormalities and normalities using the Shapiro–Wilk test. Levene test was used to check homogeneity of variance. The Student-t test was used for analysis of groups with normally distributed continuous variables. The Mann–Whitney U test was used for analysis of discrete variables, categorical variables, and continuous variables that were not normally distributed. Multiple comparisons between samples on the VAS, ODI and HADS scores were analyzed by analysis of variance (ANOVA). Multiple comparisons between samples on smoking history, diagnosis and neurologic dysfunction was analyzed by the chi-square test. Differences with two-tailed P values < 0.05 were considered statistically significant.

## Results

### Demographics of patients

According to the inclusion and exclusion criteria, 86 patients were enrolled, and 70 patients achieved at final follow-up. A total of 35 patients who underwent MISTLIF were compared with a matched cohort of 35 patients who underwent OLIF both in the L4/5 segment (Fig. 1). The mean age and gender distributions were expectedly similar between the MISTLIF and OLIF groups. The BMI in the MISTLIF group was also comparable with the OLIF group. There were no significant differences in other baseline demographic characteristics including the history of the disease, smoking history, follow-ups, and neurological function between the 2 cohorts (Table 1).

**Table 1:** Comparison of clinical features of patients in both groups.

	MISTLIF	OLIF	P value
Gender (M/F)	35 (14/21)	35 (14/21)	1.000
Age (year)	62.86 ± 8.48	64.40 ± 8.20	0.442
History (month)	46.27 ± 48.55	38.83 ± 38.71	0.481
Follow-ups (month)	16.01 ± 3.93	14.63 ± 3.01	0.106
Diagnosis			0.683
Spinal stenosis	20	23	
Spinal stenosis with instability	5	3	
Spondylolisthesis	10	9	
Smoking			0.164
Yes	4	1	
None	31	34	
Neurologic dysfunction			0.707
Sensory deficits	5	6	
Motor deficits	1	2	
Body mass index (Kg/m^2^)	24.79 ± 3.00	24.81 ± 3.55	0.979

### Perioperative characteristics of patients

OLIF group had significantly shorter bedtime, less postoperative hospital stay, less intraoperative blood loss, and less postoperative drainage, but had more times of intraoperative fluoroscopy, longer operative time, and higher total cost and high-value medical consumables cost than the MISTLIF group (Table 2).

**Table 2:** Perioperative characteristics of patients between MISTLIF and OLIF group.

	MISTLIF	OLIF	P value
Intraoperative blood loss (ml)	127.71 ± 57.40	62.86 ± 27.18	0.000
First-day drainage (ml)	128.00 ± 73.00	41.40 ± 61.60	0.000
Bedtime (days)	4.54 ± 1.67	2.51 ± 0.70	0.000
Hospital stay (days)	9.23 ± 4.70	6.17 ± 2.29	0.000
Operative time (min)	173.40 ± 32.10	205.74 ± 38.80	0.000
Fluoroscopy	19.20 ± 5.50	25.17 ± 4.62	0.000
Consumables cost (RMB)	21,653.22 ± 3492.19	32,241.22 ± 4265.85	0.000
Total cost (RMB)	44,978.39 ± 9113.57	57,794.63 ± 7602.26	0.000

### Radiographic results

Lateral X-rays showed that the PISH, IFH, and IFA after surgery were significantly increased in the OLIF group compared with those in the MISTLIF group (*P* < 0.01, Table 3). But the PISH, IFH, and IFA in the MISTLIF group were not significantly increased after the operation compared with those before the operation (*P* > 0.05, Table 4). Also, we found that the PISH, IFH, and IFA in the OLIF group were smaller than the MISTLIF group before operation (Table 5). There was no significant difference in ASC before operation between MISTLIF and OLIF group (Table 6). The ASC was increased significantly after the operation in both groups, but the ASC in the MISTLIF group was increased significantly more than the OLIF group (*P* < 0.05; Table 6).

**Table 3:** Comparison of postoperative radiological parameters between MISTLIF and OLIF group.

	MISTLIF	OLIF	P value
PISH (mm)	9.42 ± 1.94	11.94 ± 1.78	0.001
IFH (mm)	21.41 ± 2.95	23.87 ± 3.05	0.006
IFA (mm^2^)	177.07 ± 51.73	212.14 ± 51.82	0.001

**Table 4:** Changes in radiological parameters in MISTLIF group.

	Preoperation	Postoperation	P value
PISH (mm)	8.62 ± 2.43	9.42 ± 1.94	0.131
IFH (mm)	21.75 ± 2.95	21.41 ± 2.95	0.634
IFA (mm^2^)	189.06 ± 46.80	177.07 ± 51.73	0.313

**Table 5:** Comparison of preoperative radiological parameters between MISTLIF and OLIF group.

	MISTLIF	OLIF	P value
PISH (mm)	8.62 ± 2.43	6.57 ± 2.46	0.001
IFH (mm)	21.75 ± 2.95	20.15 ± 3.57	0.035
IFA (mm^2^)	189.06 ± 46.80	163.11 ± 51.18	0.030

**Table 6:** Comparison of ASC between MISTLIF and OLIF group.

	Preoperation	Postoperation	P value
MISTLIF (mm^2^)	127.45 ± 38.58	450.04 ± 66.66	0.000
OLIF (mm^2^)	137.31 ± 67.83	171.41 ± 58.55	0.028
P	0.457	0.000	

### Clinical and psychological outcomes

**Figure 4:**
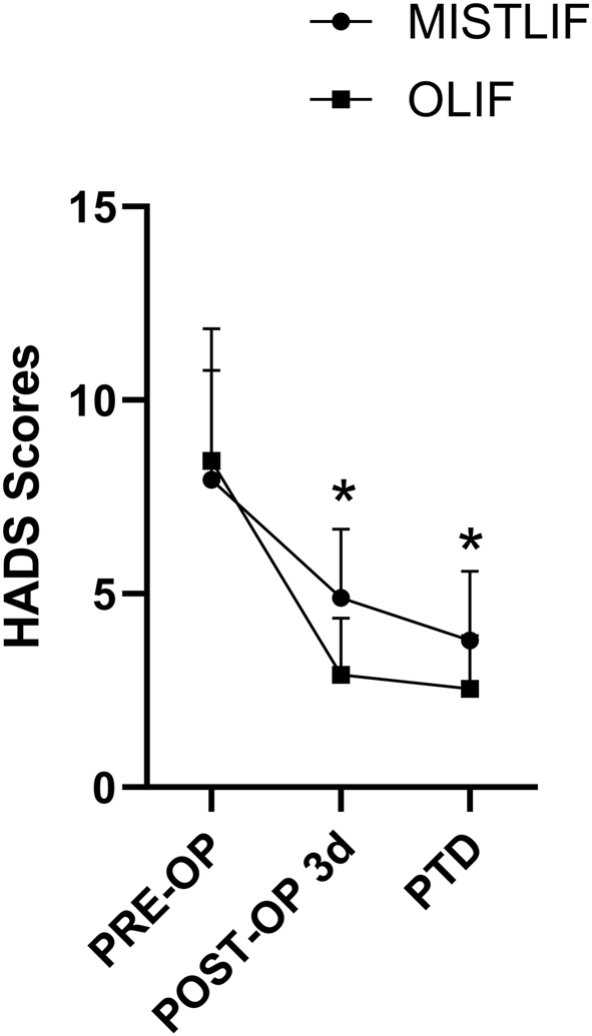
Comparison of HADS scores between MISTLIF and OLIF groups. *Indicated *P* < 0.05.

No significant differences were found in VAS scores (both lumbar and lower extremity) and ODI scores between the two groups at preoperative, postoperative 12 months, and final follow-up (*P* < 0.05, Table 7). However, the lumbar VAS scores at 1 month and 6 months after operation in the OLIF group were significantly lower than those in the MISTLIF group (Table 7). So did the ODI (Table 7). There were no significant differences in lower extremity VAS scores between the two groups at any follow-up point (*P* > 0.05, Table 7). There was no significant difference in HADS score between the MISTLIF and OLIF group preoperatively (8.43 ± 3.42 vs 7.94 ± 2.8, *P* = 0.519), which indicated that all patients felt anxious preoperatively (Fig. 4). However, the HADS scores on the postoperative day 3 (2.91 ± 1.46 vs 4.89 ± 1.78, *P* < 0.01) and PTD (2.54 ± 1.38 vs 3.80 ± 1.78, *P* < 0.01) in the OLIF group decreased significantly compared with those in the MISTLIF group (Fig. 4).

**Table 7:** Comparison between MISTLIF and OLIF before operation and follow-ups.

	MISTLIF	OLIF	P value
Lumbar VAS			
PRE-OP	2.54 ± 2.03	2.80 ± 1.95	0.591
Post-OP 1 mon	2.34 ± 0.84	1.89 ± 0.87	**0.028**
Post-OP 6 mon	1.80 ± 0.99	1.23 ± 0.97	**0.018**
Post-OP 12 mon	1.17 ± 0.75	1.06 ± 0.76	0.529
Final follow-up	1.09 ± 0.80	0.91 ± 0.77	0.362
Leg VAS			
PRE-OP	5.17 ± 1.74	4.69 ± 1.25	0.185
Post-OP 1 mon	2.03 ± 1.12	1.97 ± 1.15	0.834
Post-OP 6 mon	1.34 ± 0.68	1.20 ± 0.63	0.367
Post-OP 12 mon	1.17 ± 0.86	1.14 ± 0.77	0.884
Final follow-up	0.97 ± 0.75	0.80 ± 0.68	0.318
ODI			
PRE-OP	58.51 ± 20.41	62.06 ± 20.23	0.468
Post-OP 1 mon	23.14 ± 5.91	20.17 ± 4.27	**0.019**
Post-OP 6 mon	19.66 ± 5.28	16.40 ± 5.15	**0.011**
Post-OP 12 mon	17.66 ± 4.86	14.97 ± 4.64	0.216
Final follow-up	15.31 ± 3.36	14.11 ± 3.91	0.212

Significant values are in [bold].

### Complications

Considering complications, 8 patients (22.86%, 8/35) were found in the OLIF group, including 7 patients with transient thigh flexion weakness and 1 patient with segmental artery injury. While 6 patients (17.14%, 6/35) with complications were found in the MISTLIF group, including 1 patient with the dural tear, 5 patients with paresthesia or radiculitis. Paresthesia or radiculitis was significantly relieved after treatment with nerve nutrition, dehydration, and hormones.

## Discussion

Preserving the soft tissue envelope and understanding the anatomy and biology of the posterior spinal musculature are key concepts in minimally invasive spine surgery^14^. Based on this concept, the minimally invasive treatment of DLSS is applied. Many scholars have reported that MISTLIF through bilateral decompression via unilateral approach has achieved good results in the treatment of DLSS^15–17^. OLIF is a minimally invasive anterior retroperitoneal approach surgery, which has been very popular in recent years. In OLIF surgery, the surgeon reaches the operative segment through the anatomical space between the abdominal aorta and the psoas muscle via the retroperitoneal space and preserves the integrity of the posterior structure of the spine. In this study, OLIF shows more advantages in the treatment of single-segment lumbar spinal stenosis than MISTLIF.

OLIF had less bleeding and less postoperative drainage than MISTLIF as reported previously^18^. The possible reasons are as follows: (1) The OLIF passes through the retroperitoneal approach to the disc, with few peripheral vessels, which are easily protected under direct vision. (2) MISTLIF needs to open the spinal canal, which can easily damage the venous plexus and cause bleeding; especially when contralateral decompression is performed, effective hemostasis is difficult. (3) Bony structural damage is avoided since laminectomy is not required in OLIF. The decrease in the incidence of iatrogenic disturbance to the surrounding tissues and nerves ultimately yields better outcomes in surgical bleeding^19^. The shorter postoperative hospital stay and bedtime in the OLIF group were closely related to less intraoperative injury and less postoperative drainage, which was controversial in previous studies^20,21^. Pain caused by injury to paravertebral muscles and articular processes in the MISTLIF group was also responsible for increased postoperative hospital stay and bedtime. However, the times of fluoroscopy were significantly increased in the OLIF group than those in the MISTLIF group. Possible reasons are as follows: (1) Repeated fluoroscopy is required to determine the orientation and location of the cage during the OLIF procedure. (2) Spine surgeons are familiar with posterior surgery and unfamiliar with lateral surgery. Therefore, the lack of OLIF surgical experience is also the reason for the increase in fluoroscopy times. In contrast to previous reports^22,23^, the intraoperative time in the OLIF group was longer than in the MISTLIF group. The possible reasons are as follows: (1) Time of repositioning and disinfection in the OLIF procedure was also included in the intraoperative time in our study. (2) The repeated intraoperative fluoroscopy, surgeon’s expertise, and repertoire were the other related reasons. Therefore, to avoid occupation of time in changing the patient’s position, repreparing and redrapping the surgical field, it has been reported that posterior fixation is performed under the same position. Blizzard reported that the mean operative time of single-position (SP) LLIF (lateral lumbar interbody fusion) or OLIF with bilateral pedicle screw fixation was 87.9 min (range: 49–195 min)^24^. According to a study comparing single-position versus dual-position (DP) surgery for pedicle screw fixation and lateral interbody fusion, SP surgery could reduce the average surgery time by about 31 min^25^. A recent meta-analysis also showed that SP surgery in LLIF (including OLIF technique) combined with pedicle screw fixation significantly shortened operative time compared with DP LLIF^26^. Thus, enhanced the surgeon’s training of posterior fixation in single position can significantly improve surgical efficiency. Higher cost in the OLIF group is mainly related to high-value medical consumables (Cage and artificial bone). This shortcoming may limit the widespread use of OLIF in the treatment of DLSS.

The most important treatment of DLSS is decompression by increasing the area of the spinal canal. OLIF is an indirect decompression procedure, whereas MISTLIF is a direct decompression procedure. In our study, both achieved effective decompression. In this study, although the preoperative PISH in the OLIF group was smaller than that in the MISTLIF group, the postoperative PISH was found significantly larger in the OLIF group. The effective increment and maintenance of IFH and IFA in the OLIF group were closely related to the increment of PISH. The possible reasons are as follows: (1) The cage, which was inserted into the disc gap through the Kambin triangle in the MISTLIF procedure, is smaller than that in the OLIF procedure. It is reported that the triangle between the exiting and traversing nerve roots above the superior margin of the inferior pedicle is narrow. The triangle area is from 1.83 to 2.19 cm^2^^27^. If the cage was large in the MISTLIF procedure, the never roots were easy to injure. Thus the cage was confined by the anatomy of the Kambin triangle and the PISH in the MISTLIF group was not larger enough. (2) In MISTILF surgery, only one side of the facet joints was resected while the contralateral side was usually preserved, so the intervertebral space may not be effectively expanded, especially for patients with facet osteophytosis^28^. Although there was no significant increase in PISH, IFH, and IFA in the MISTLIF group, the postoperative spinal canal area was significantly larger than that in the OLIF group, reflecting the advantage of direct decompression.

Postoperative pain relief and lumbar function recovery were the key focus of both the surgeons and patients. In our study, VAS and ODI scores were significantly decreased, especially in the first 6 months. The symptoms were further improved over time in the follow-up periods and reached a steady state. However, the lumbar VAS and ODI scores at 1 month and 6 months after operation in the OLIF group were significantly lower than those in the MISTLIF group, which was consistent with Kim’s report^10^. The reasons are as follows: (1) The paraspinal muscles are not separated from the bony structure, and there is no damage to the posterior branch of the nerve, which reduces the denervation or fatty degeneration of the paraspinal muscles, thereby reducing the incidence of lower back pain in the OLIF group^5,29^. (2) There was little iatrogenic damage to nerves and tissues around the spinal canal in the OLIF group^30^. Although there was no significant difference in VAS and ODI scores of the lower extremity, we found that there were more patients with transient thigh flexion weakness in the OLIF group. This was likely due to genitofemoral nerve disturbance during the procedure, in which the disturbance is usually temporary and reversible. Both the surgeon's skill and the patient's mental health influence the clinical outcome. The quality of life of patients with lumbar spinal stenosis is closely related to their emotional status^31^. It is reported that anxiety status after spinal surgery could lead to poor clinical outcomes^32^. Pain is considered a psychosomatic factor that connects both the physical and psychological domains^33^. It was reported by the previous studies that poor psychological outcomes were correlated with elevated pain scores and lower physical function^34,35^. In a recent study, it was reported that the postoperative HADS scores were positively correlated with the low back pain VAS scores^36^. At the same time, depression can also negatively influence rehabilitation after surgery^37^. In my opinion, there is a bidirectional relationship between depression and functional disability. In our study, the HADS scores on the postoperative day 3 and PTD in the OLIF group decreased significantly compared with those in the MISTLIF group. This may be related to the lower lumbar VAS scores after operation in the OLIF group. In addition to pain, other factors may also affect the psychological state of patients after surgery, such as early getting out of bed, drainage, etc^38^. Shorter bedtime and hospital stay after operation, less intraoperative blood loss, and postoperative drainage in the OLIF group would be other reasons.

This study also has some limitations. First, it is a retrospective cohort study, a multi-center study needs to be considered to decrease the bias. Second, the surgeons and patients had a different understanding of prognosis and treatment, which may affect the evaluation of results and cause bias. Third, the small sample size and short follow-up period may have affected the strength of the statistical analysis. Although OLIF could acquire good indirect decompression efficacy, it is still necessary to extend the follow-up period to confirm this conclusion because the spinal canal could remodel during long-term follow-up after surgery^39^. A high-quality study of a large sample size and long-term follow-up period is still needed to compare the results of OLIF and MISTLIF.

## Conclusion

Both OLIF and MISTLIF with bilateral decompression via unilateral approach are effective treatments for mild to moderate symptomatic DLSS. While both OLIF and MISTLIF provide optimal clinical outcomes, in comparison to an age-, sex-, and segment-matched MISTLIF cohort, OLIF showed the advantages of less surgical invasion, lower incidence of postoperative low back pain, faster postoperative recovery, and less anxiety in single-level DLSS. Regardless of cost, OLIF seems to be a better option to treat mild to moderate symptomatic DLSS in modern days.

## Data Availability

The datasets analysed during the current study are not publicly available due privacy of the patients but are available from the corresponding author on reasonable request.
